# Facilitating fine-grained intra-urban dengue forecasting by integrating urban environments measured from street-view images

**DOI:** 10.1186/s40249-021-00824-5

**Published:** 2021-03-25

**Authors:** Kang Liu, Ling Yin, Meng Zhang, Min Kang, Ai-Ping Deng, Qing-Lan Li, Tie Song

**Affiliations:** 1 Shenzhen Institute of Advanced Technology, Chinese Academy of Sciences, Shenzhen, 518055 People’s Republic of China; 2 Beijing Key Laboratory of Urban Spatial Information Engineering, Beijing, 100038 People’s Republic of China; 3 Guangdong Provincial Center for Disease Control and Prevention, Shenzhen, 511430 People’s Republic of China

**Keywords:** Dengue forecasting, Intra-urban, Fine-grained, Urban environment, Street-view image

## Abstract

**Background:**

Dengue fever (DF) is a mosquito-borne infectious disease that has threatened tropical and subtropical regions in recent decades. An early and targeted warning of a dengue epidemic is important for vector control. Current studies have primarily determined weather conditions to be the main factor for dengue forecasting, thereby neglecting that environmental suitability for mosquito breeding is also an important factor, especially in fine-grained intra-urban settings. Considering that street-view images are promising for depicting physical environments, this study proposes a framework for facilitating fine-grained intra-urban dengue forecasting by integrating the urban environments measured from street-view images.

**Methods:**

The dengue epidemic that occurred in 167 townships of Guangzhou City, China, between 2015 and 2019 was taken as a study case. First, feature vectors of street-view images acquired inside each township were extracted by a pre-trained convolutional neural network, and then aggregated as an environmental feature vector of the township. Thus, townships with similar physical settings would exhibit similar environmental features. Second, the environmental feature vector is combined with commonly used features (e.g., temperature, rainfall, and past case count) as inputs to machine-learning models for weekly dengue forecasting.

**Results:**

The performance of machine-learning forecasting models (i.e., MLP and SVM) integrated with and without environmental features were compared. This indicates that models integrating environmental features can identify high-risk urban units across the city more precisely than those using common features alone. In addition, the top 30% of high-risk townships predicted by our proposed methods can capture approximately 50–60% of dengue cases across the city.

**Conclusions:**

Incorporating local environments measured from street view images is effective in facilitating fine-grained intra-urban dengue forecasting, which is beneficial for conducting spatially precise dengue prevention and control. 
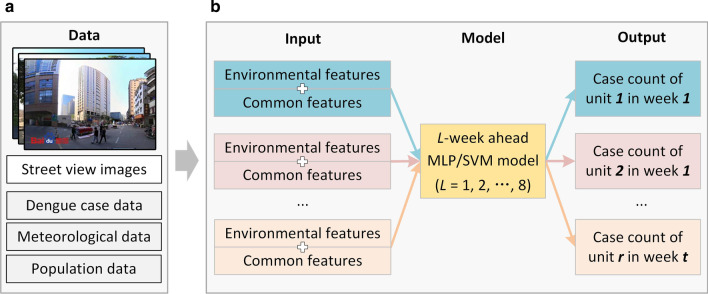

## Background

Dengue fever (DF) is an acute infectious disease caused by infection with any one of the four serotypes of dengue virus (DENV 1–4) transmitted by *Aedes* mosquitoes [1, 2]. In recent years, mosquito-borne infectious diseases have spread in tropical and subtropical urban areas and have become a serious global public health problem. In the absence of a vaccine, disease surveillance and mosquito control are the primary preventive measures for controlling the spread of the disease [3]. Against this background, dengue forecasting at a fine-grained intra-urban scale is urgently required to guide prevention and control.

Existing studies have proven that mosquito dynamics are sensitive to changes in meteorological conditions [4–6]. For instance, temperature and humidity are known to influence longevity, feeding behavior, mating, and oviposition of mosquito vectors, while rainfall contributes to the generation of vector breeding sites [7, 8]. For these reasons, meteorological factors have become the primary independent variables in dengue forecasting [3, 9–12]. For instance, Hii et al. developed a weather-based dengue forecasting model that warns against a dengue epidemic 16 weeks in advance [11]. They stated that models using temperature and rainfall could be simple, precise, and low-cost tools for dengue forecasting and prevention. Sang et al. found that imported dengue cases in the previous month, the monthly minimum temperature in the previous month, and monthly accumulative precipitation with three-month lags could anticipate dengue outbreaks one month in advance [12].

However, weather is not the only necessary condition for mosquito breeding. Some local environments provide more ideal conditions for mosquito breeding and survival than others, even under the same suitable weather conditions [13, 14]. Intuitively, dirty roadsides or building sites would provide more breeding grounds for mosquitoes than well-maintained places. Existing studies have also found that urban villages with poor sanitation, overcrowded population and buildings, and pot-holed roads, usually provide high environmental suitability for mosquito vectors [14]. Some street-view elements also function as potential mosquito breeding grounds, such as dustbins, water, flowerpots, trucks and sand (indicating that there may be building sites around) [13, 15]. Theoretically, integrating the physical environment as an important factor, together with other commonly used factors, would improve dengue forecasting performance, especially in fine-grained intra-urban settings.

How to quantitatively depict urban physical environments is a key question. Traditionally, the quality of physical environments in urban areas has been assessed through questionnaire surveys [16] and field audits [17, 18]. Such data acquisition methods are expensive and time-consuming. The collected datasets are usually small and biased, as they are influenced by the attitudes, skills, or other subjective factors of the participants. The easy availability and widespread use of remotely sensed imagery simplifies the measurement of the overall greenness of a given area using the normalized difference vegetation index (NDVI), which is much more objective, efficient, and economical [19–21]. However, NDVI only depicts a single perspective (i.e., greenness) of the urban environment, and the overhead-view greenery measured by NDVI often differs from the eye-level greenery perceived by people, as NDVI may fail to detect lawns or shrubs under tree canopies, green walls, or vegetation covered by bridges [22, 23].

In recent years, emerging street views, which are electronic maps based on actual scenery, provide free and rich data sources for assessing the human-perceived urban landscape [24]. The rapid development of deep learning techniques has also greatly promoted the value and applicability of the data [25–28]. Several large internet companies, such as Google, Baidu, Tencent, and Microsoft, have launched online street-view services. Street-view images of various locations in cities can be conveniently retrieved and downloaded through application programming interfaces (APIs) of these online map service providers. Using street-view images, a wide range of urban studies have been conducted including 3D city reconstruction [29], urban scene recognition [25, 26, 30, 31], route selection [32], and urban function recognition [33]. In particular, as visual perception of landscape serves as a basis for urban planning and quality of life, several studies have evaluated landscape qualities [34–37] and human perceptions of urban appearance [38–40], while investigating their associations with socioeconomic factors [41–43], physical activity [44, 45], street accessibility [33], and other outcomes. More importantly, poor street quality and some specific environmental elements (e.g., trees, plants, dustbins, flowerpots, sand, and water) that imply potential mosquito breeding grounds can be conveniently measured and identified from street-view images [13, 37]. A recent study also demonstrated that street-view images can estimate the dengue incidence rate at various locations [46, 47].

Therefore, this study proposes a novel strategy to integrate urban environments measured from street-view images into fine-grained intra-urban dengue forecasting processes. Specifically, a pre-trained PSPNet model (trained on the image segmentation dataset ADE20K) was applied to extract the outdoor scene features from the street-view images. Feature vectors of images collected from the same unit were then averaged as an environmental feature vector for the unit. Then, the environmental feature vectors were combined with the commonly used features (e.g., temperature, rainfall, past cases) to enhance the supervised learning-based dengue forecasting models, such as the multilayer perceptron (MLP) and support vector machine (SVM) used in our study. The effectiveness of the proposed dengue forecasting approach was tested on the most threatened Chinese city, Guangzhou, at the township level.

## Methods

### Study area and data

**Fig. 1:**
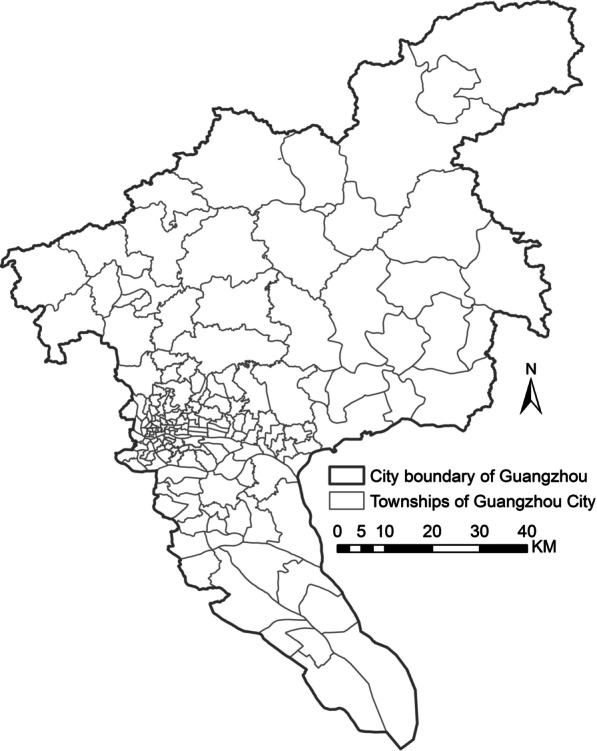
Study area. All 167 townships in Guangzhou City, China

Guangzhou is a first-tier city located in South China (Fig. 1), having an area of 7434 km^2^ and comprising over 15 million permanent residents until the end of 2019. The climate of Guangzhou is humid and subtropical, with high temperatures and humidity in summer and comparatively mild and dry weather in winter. The suitable climate, large floating population, and close proximity to Southeast Asia where dengue has been hyperendemic for decades, renders Guangzhou the most threatened city in China [48–50].

As shown in Fig. 1, the 167 townships of Guangzhou were used as urban units in this study for fine-grained intra-urban dengue predictions. According to statistics, approximately 20% of townships have areas of less than 2 km^2^, and 37% have less than 5 km^2^. The prediction units in this study are much smaller than those in the existing studies.

### Dengue case data

**Fig. 2:**
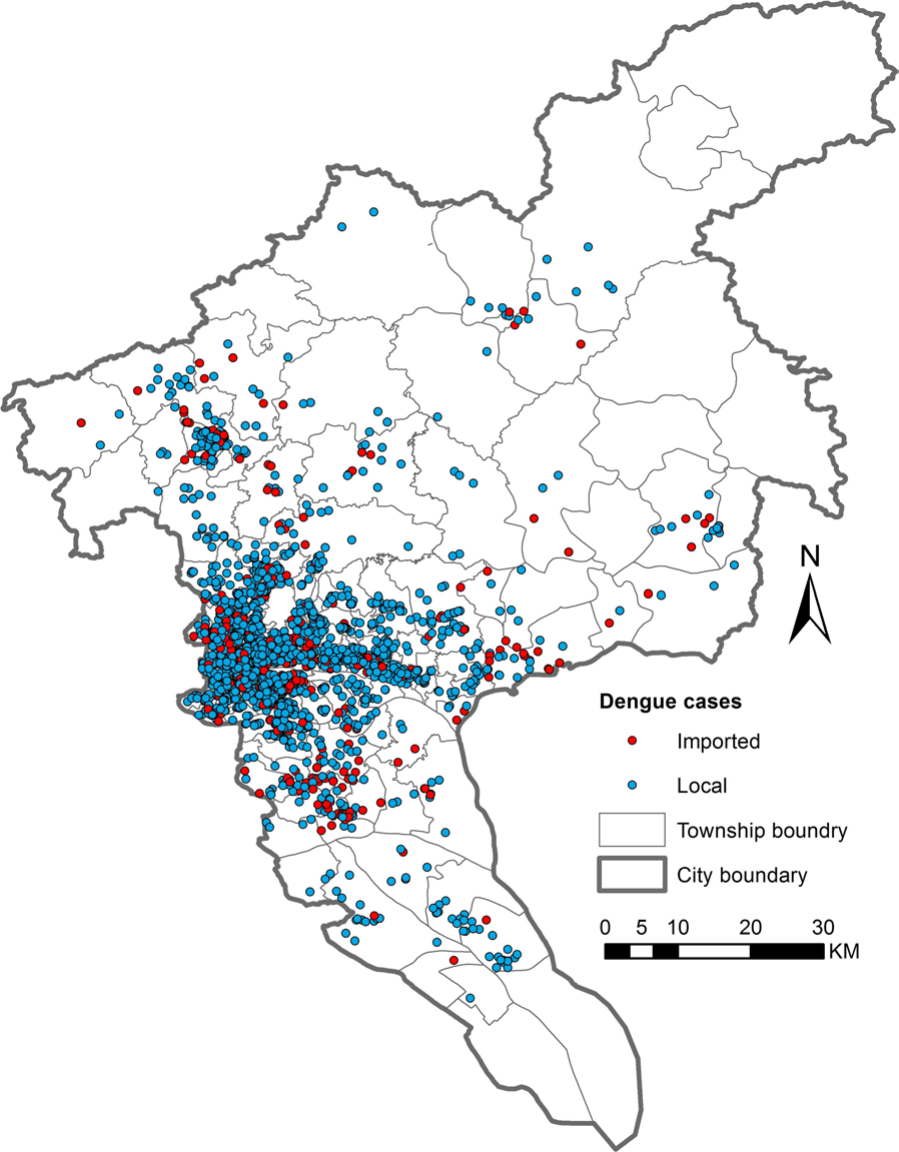
Spatial distribution of dengue cases in Guangzhou City, China between January 2015 and September 2019

Guangzhou experienced its worst dengue epidemic in 2014, with an incidence of 37 445 locally acquired dengue cases exceeding the historical average by two orders of magnitude [49]. Since 2015, Guangdong Province and Guangzhou City have spared no effort to prevent and control dengue epidemics, reducing dengue cases in recent years. To establish prediction models under the current control policy, this study used dengue case data for Guangzhou City between January 1, 2015, and September 22, 2019, provided by the Guangdong Center for Disease Control and Prevention (Guangdong CDC). The attributes of each anonymous case included residential address, onset date, and type (imported or local). Residential addresses of the cases were converted to geographic coordinates using the geocoding API of Baidu Maps [51]. Figure 2 shows the spatial distribution of dengue cases in Guangzhou City during the study period.

**Fig. 3:**
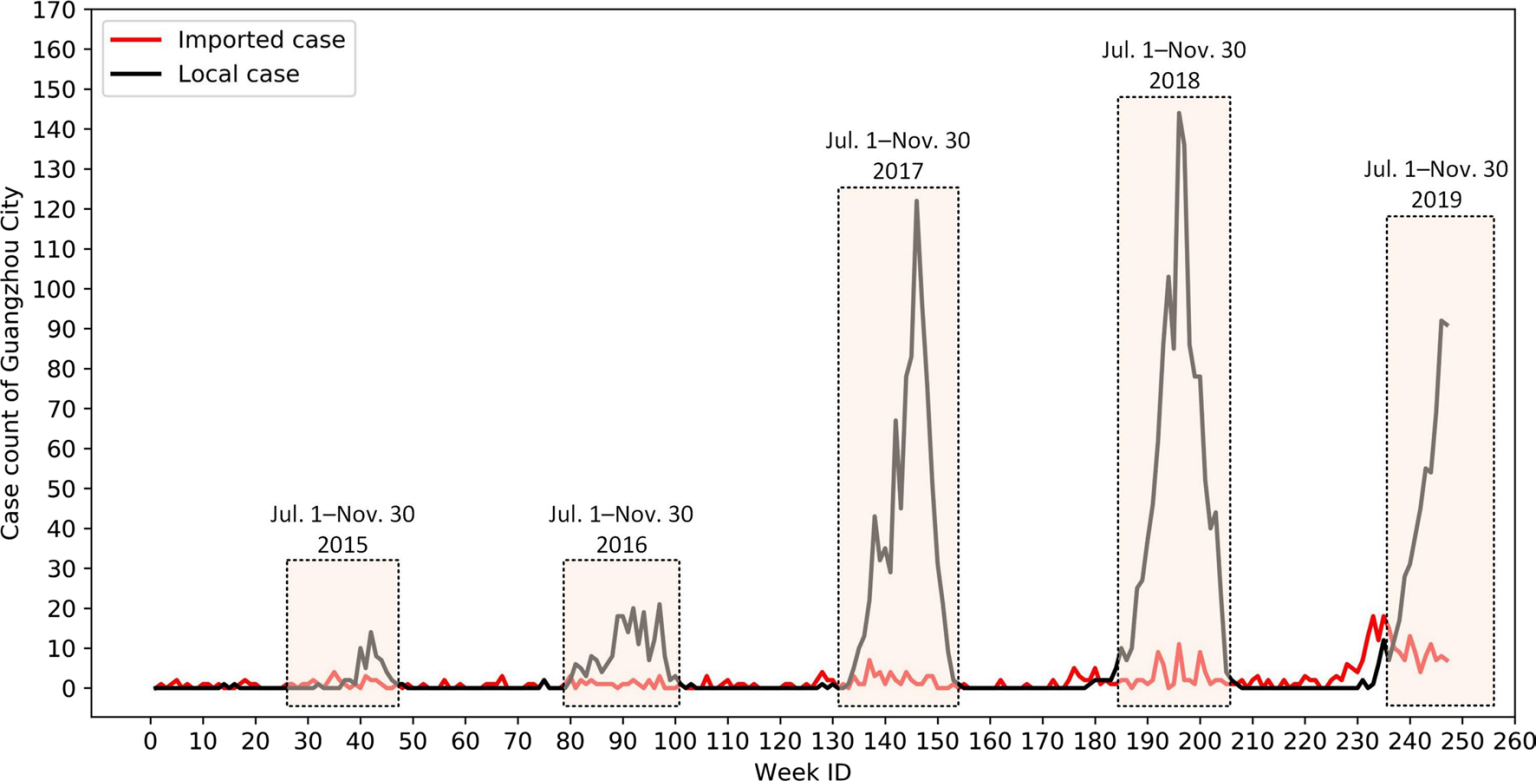
Weekly dengue case count of Guangzhou City from January 2015 to September 2019

We then counted the dengue cases inside each township by week based on the onset date. Figure 3 presents the weekly imported and local case counts of the city during the study period. According to Fig. 3, we identified July 1 to November 30 as the concentrated outbreak period, when the number of dengue cases was apparently above normal.

### Meteorological data

**Fig. 4:**
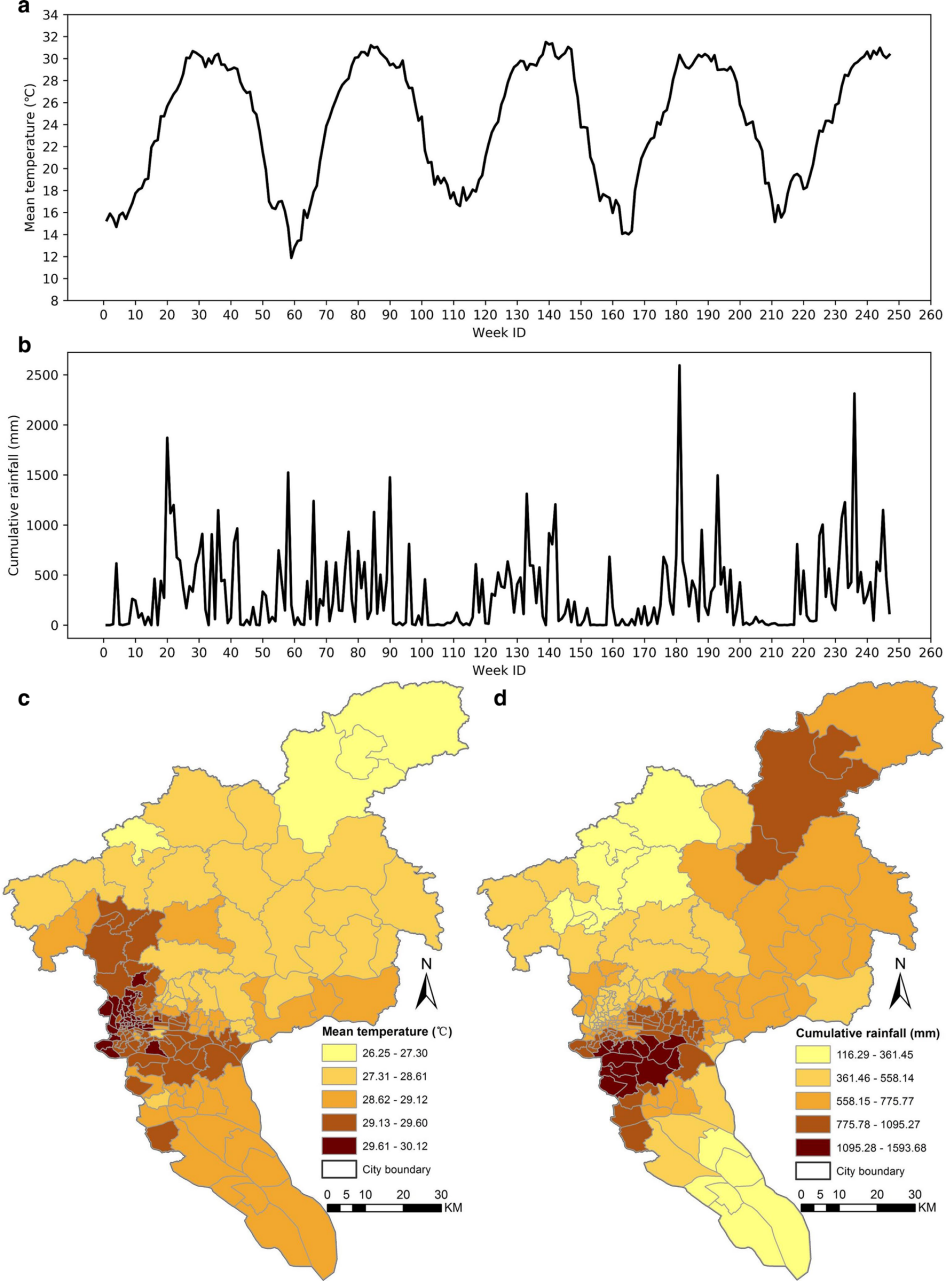
Meteorological data of Guangzhou City. **A** Weekly mean temperature and **B** weekly cumulative rainfall of a township from January 2015 to September 2019. **C** Weekly mean temperature and **D** weekly cumulative rainfall of all townships within Guangzhou City during the week of September 12–18, 2016

The daily mean temperature and daily rainfall recorded by nearly 300 weather stations in Guangzhou during the study period were obtained from the Guangdong Meteorological Bureau. The station-based data were spatially interpolated to a fine resolution using the ordinary Kriging method, and then averaged (for temperature) or summed (for rainfall) at the township level. Figures 4A, B illustrate the weekly mean temperature and cumulative rainfall of one arbitrarily selected township during the study period. Figures 4C, D show the weekly mean temperature and cumulative rainfall of all townships within the city during an arbitrarily selected week (i.e., September 5–11, 2016).

### Population data

The population data of Guangzhou used in this study were obtained from the well-known open data source, WorldPop [52, 53]. However, WorldPop provides yearly population datasets from 2015 to 2019, which cannot meet the time interval (weekly) of dengue prediction. Considering that Guangzhou is a well-developed city and its spatial population distribution pattern has not changed substantially in recent years, we used the population dataset of 2017 to represent the population from 2015 to 2019.

**Fig. 5:**
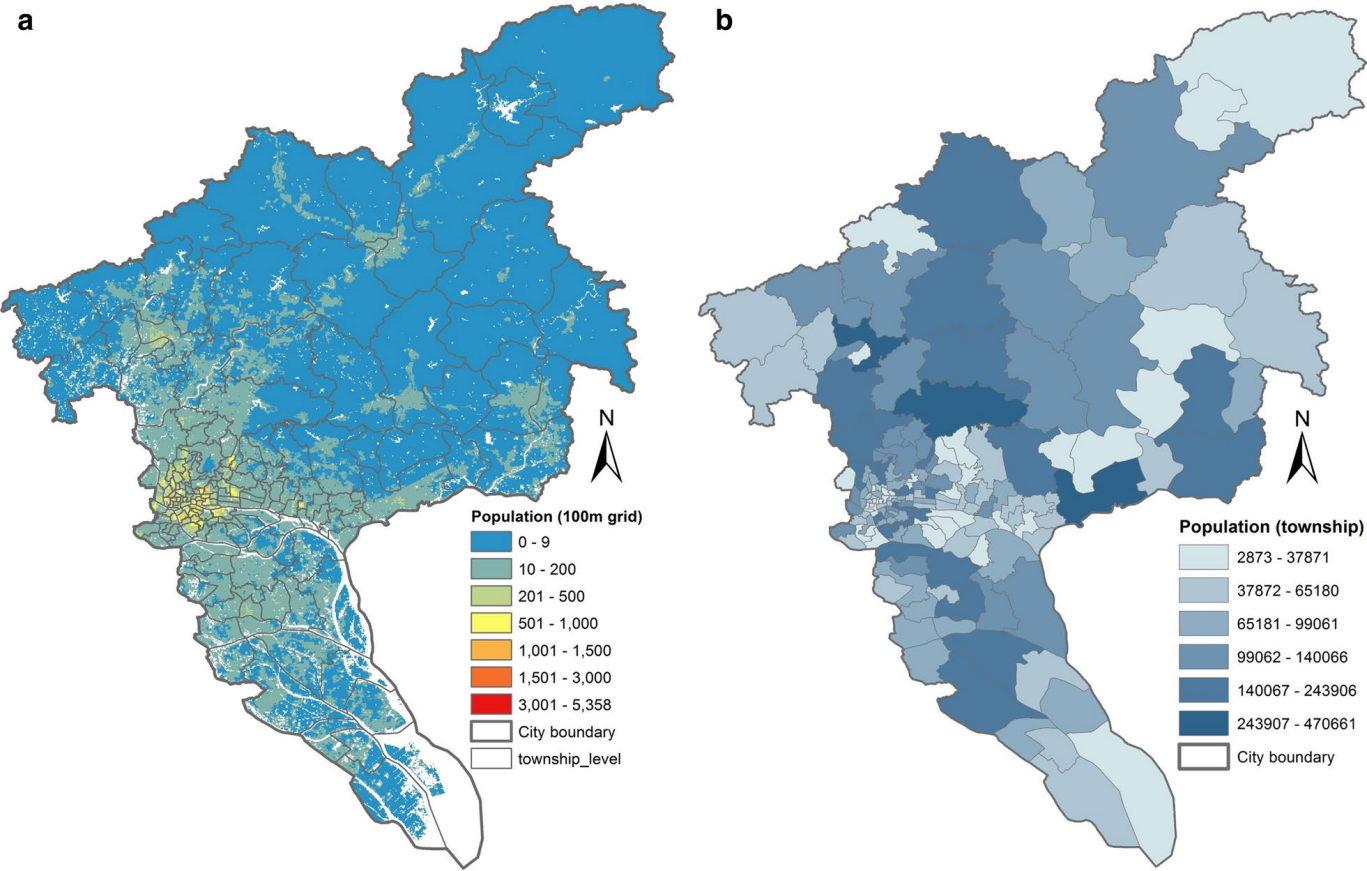
Population data (2017) of Guangzhou City. **A** 100-m gridded population counts provided by the WorldPop. **B** Township-based population aggregated from the 100 m-grid population counts

As shown in Fig. 5, the 100-m gridded population data for 2017 were aggregated at the township level. A township with a larger population implies more hosts for the mosquito vectors and the incidence rate is more likely to be higher.

### Street view images

The street-view images used in this study were obtained from Baidu Maps using the Web Service API [54]. The corresponding street-view image at the location can be obtained using the coordinates (i.e., longitude and latitude) and setting the field of view (i.e., the parameter “fov”), horizontal view angle (i.e., the parameter “heading”), image size (i.e., the parameters “width” and “height”), and developer key (i.e., the parameter “ak”).

The URL format for requesting an image under specific parameters is http://api.map.baidu.com/panorama/v2?ak=ReplaceYourDeveloperKey&width=400&height=300&location=113.25529,23.11419&fov=90&heading=0. Note that one needs to apply and use her/his developer key to open the example URL.

**Fig. 6:**
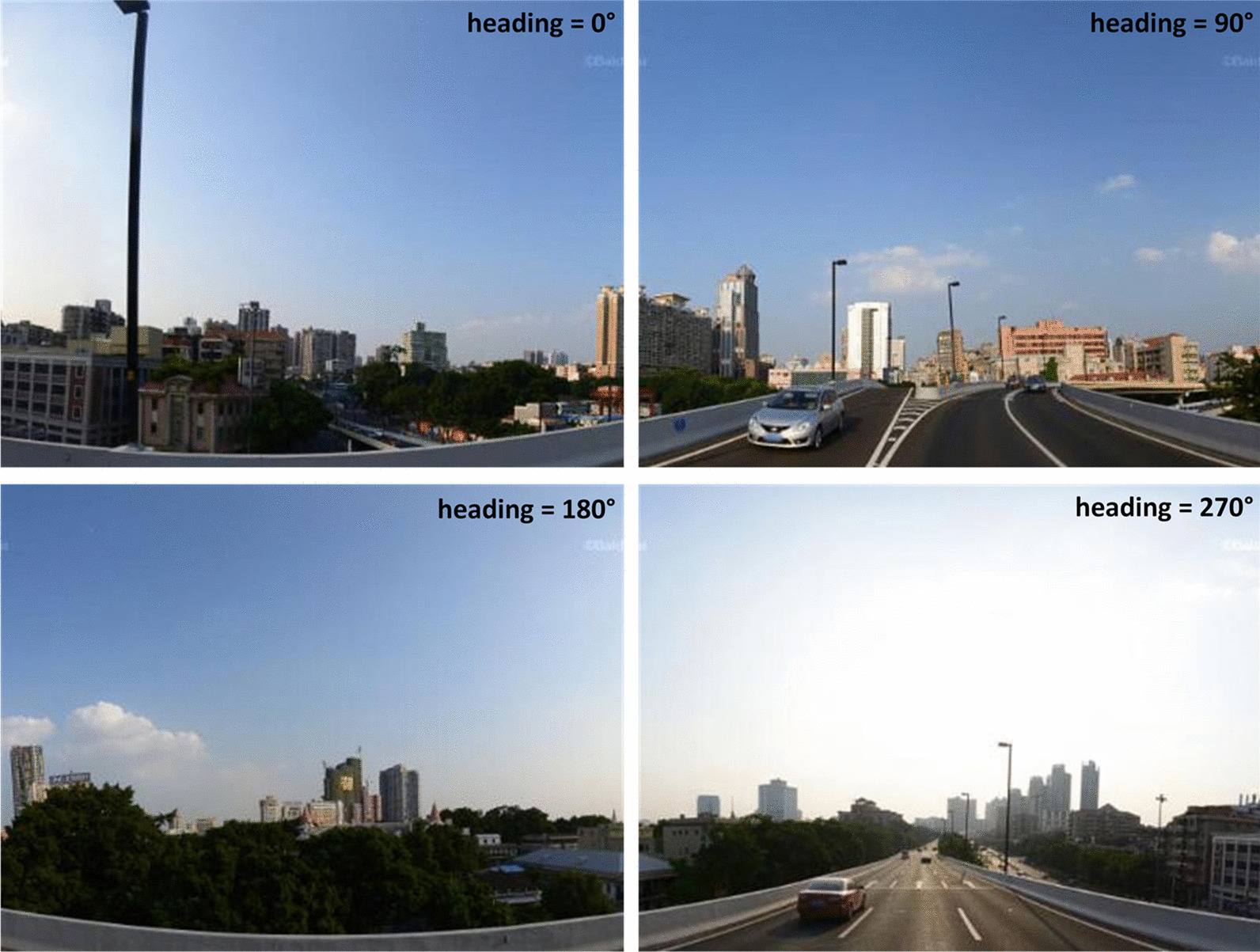
Street-view images acquired at a location with heading degrees of 0, 90, 180, and 270

As shown in Fig. 6, by setting the parameter “heading” as 0, 90, 180, and 270 degree, we can obtain four street-view images of a given location. Those images comprehensively depict the physical environment of the location.

Street-view images were acquired at a distance interval of 150 m along OpenStreetMap (OSM) [55] streets within Guangzhou City. Considering that street-view images collected in areas with few or no population are useless for depicting human living environments, we only retained images collected from spatial units (i.e., 100-m grids) with a population of no less than 10 (please refer to Fig. 5A). Thus, four street-view images can be acquired at each location (if the request is successful), resulting in 112 447 total images (512 × 512 pixels) across the city.

### Method framework and steps

**Fig. 7:**
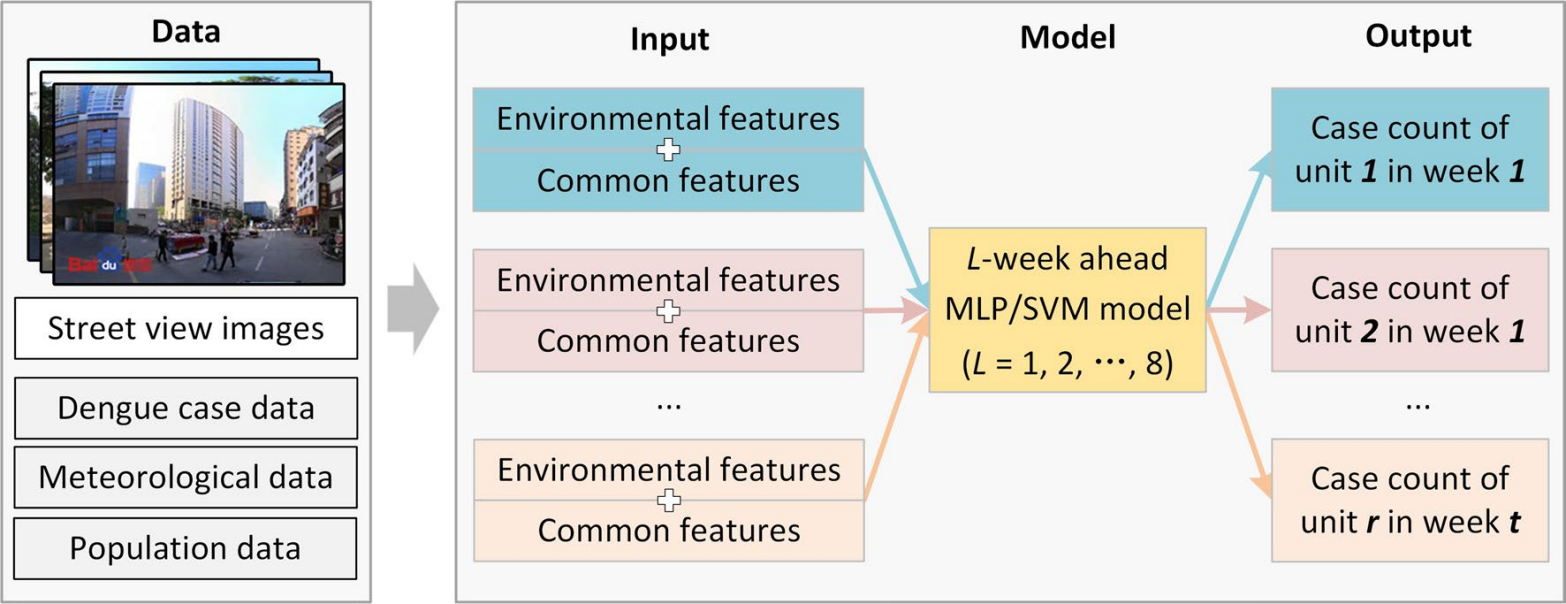
Framework of the proposed approach

The framework of our methodology is illustrated in Fig. 7. To facilitate fine-grained intra-urban dengue forecasting, environmental features were extracted from street-view images and then combined with the commonly used features (i.e., past cases, temperature, rainfall, and population) as inputs to the forecasting models (i.e., MLP and SVM). Models for *L*-week (*L* = 1, 2,…, 8) ahead forecasting were separately trained and applied.

The processes of environmental feature extraction, common feature extraction, forecasting model construction, and forecasting performance evaluation are introduced in detail in the following sections.

### Environmental feature extraction

**Fig. 8:**
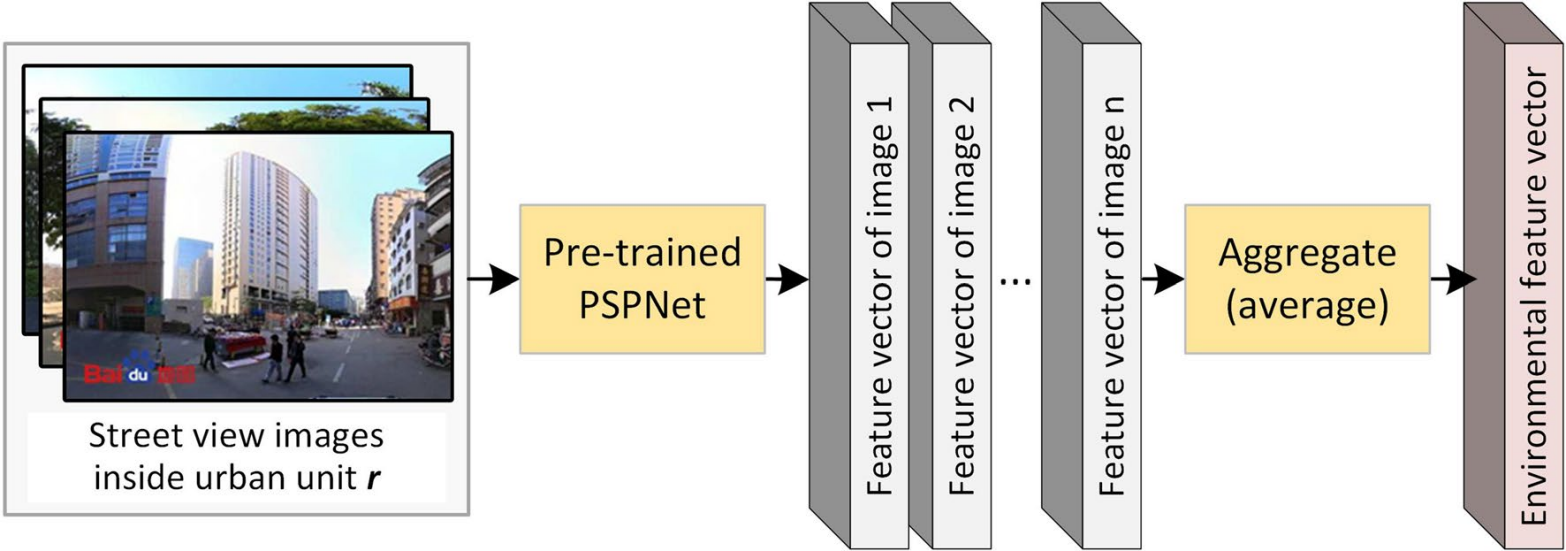
Extracting the environmental feature vector of an urban unit from street-view images using the pre-trained PSPNet model

As shown in Fig. 8, this study applied a pre-trained deep convolutional neural network called PSPNet [56] to extract the feature vector of each street-view image. The environmental features of each urban unit were then obtained by aggregating the image feature vectors collected within the unit.

The PSPNet provides a superior framework for pixel-level scene-parsing tasks (i.e., labeling the category of all pixels within the image), and received the champion of the ImageNet Scene Parsing Challenge in 2016 [57]. As we mainly focused on outdoor scenes, this study used a PSPNet model pre-trained on the ADE20K dataset [57], which included numerous images with 150 indoor and outdoor scene categories. Feeding a street-view image into the pre-trained PSPNet, each image pixel can be labeled with one of the 150 scene categories. Based on the pre-trained model, we calculated the pixel proportions of the 64 selected outdoor scenes for each image [26]; thus, a 64-dimensional feature vector could be constructed to describe the image.

**Fig. 9:**
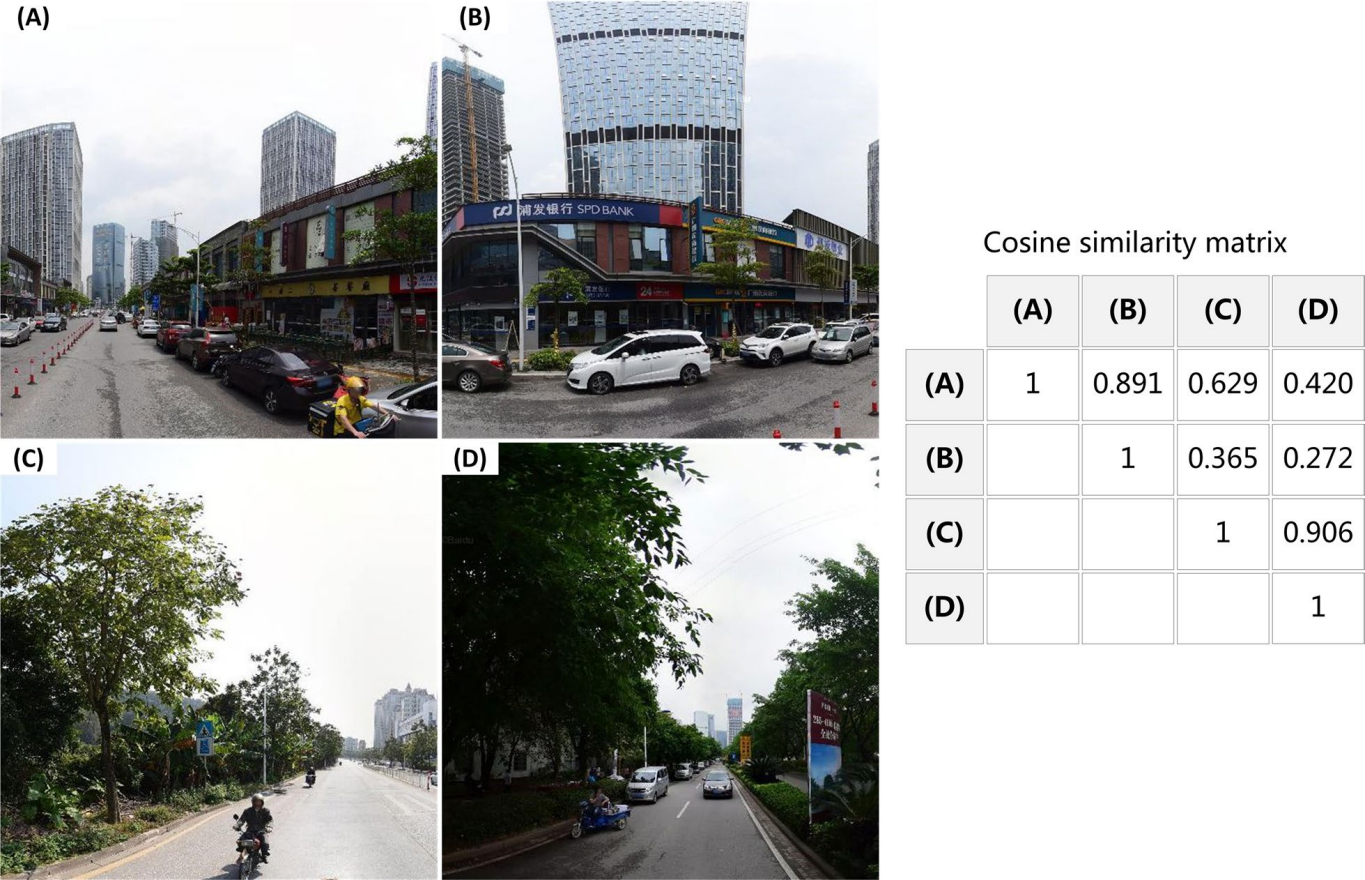
Cosine similarity matrix of four street-view images measured by their feature vectors extracted from the pre-trained PSPNet model

As a demonstration, Fig. 9 shows the cosine similarity matrix of four street-view images measured by their 64-dimensional feature vectors. We can see that street-view images with more similar scene elements would have more similar feature vectors [such as images (A) and (B) and images (C) and (D)], indicating that the extracted feature vectors can successfully depict physical environments at various places.

Finally, feature vectors of street-view images collected within the same urban unit were averaged to be a 64-dimension environmental feature vector to represent the physical environment of the unit (i.e., township in our study). Thus, urban units with similar physical settings would have similar environmental features. We averaged the feature vectors of all inside images to represent the entire township because most of the townships are very small, and the population in a few larger townships is concentrated. If the spatial units were very large with a dispersed population distribution, the averaged features would have less representativeness.

### Common feature extraction

This study used epidemical, meteorological, and sociodemographic variables as common features, which have been widely used and proven as important factors for dengue forecasting in previous studies [3, 9–12]. As shown in Table 1, 11 common features were extracted for each urban unit (i.e., township) from past cases (including imported and local cases), mean temperature, cumulative rainfall, and population. Epidemical and meteorological factors are dynamic features with time lags of up to four weeks, while the population factor is a static feature.

**Table 1:** Common features extracted for each urban unit

No	Category	Feature
1	Epidemical factor (dynamic)	Case count of the $(t-1)$-th week
2		Case count of the $(t-2)$-th week
3		Case count of the $(t-3)$-th week
4		Case count of the $(\mathrm{t}-4)$-th week
5		Case count of past four weeks
6	Meteorological factor (dynamic)	Mean temperature of the $(t-1)$-th week
7		Mean temperature of the $(t-2)$-th week
8		Mean temperature of the $(t-3)$-th week
9		Mean temperature of the $(t-4)$-th week
10		Cumulative rainfall of past four weeks
11	Sociodemographic factor (static)	Population

### Forecasting model construction

This study used SVM and MLP as basic dengue forecasting models, which have been widely used in the existing literature [1]. Specifically, a linear kernel was used in the SVM-based regression model. For the MLP-based regression model, we used one hidden layer of 100 neurons, applied “tanh” as the activation function, and set the learning rate to 0.001.

**Fig. 10:**
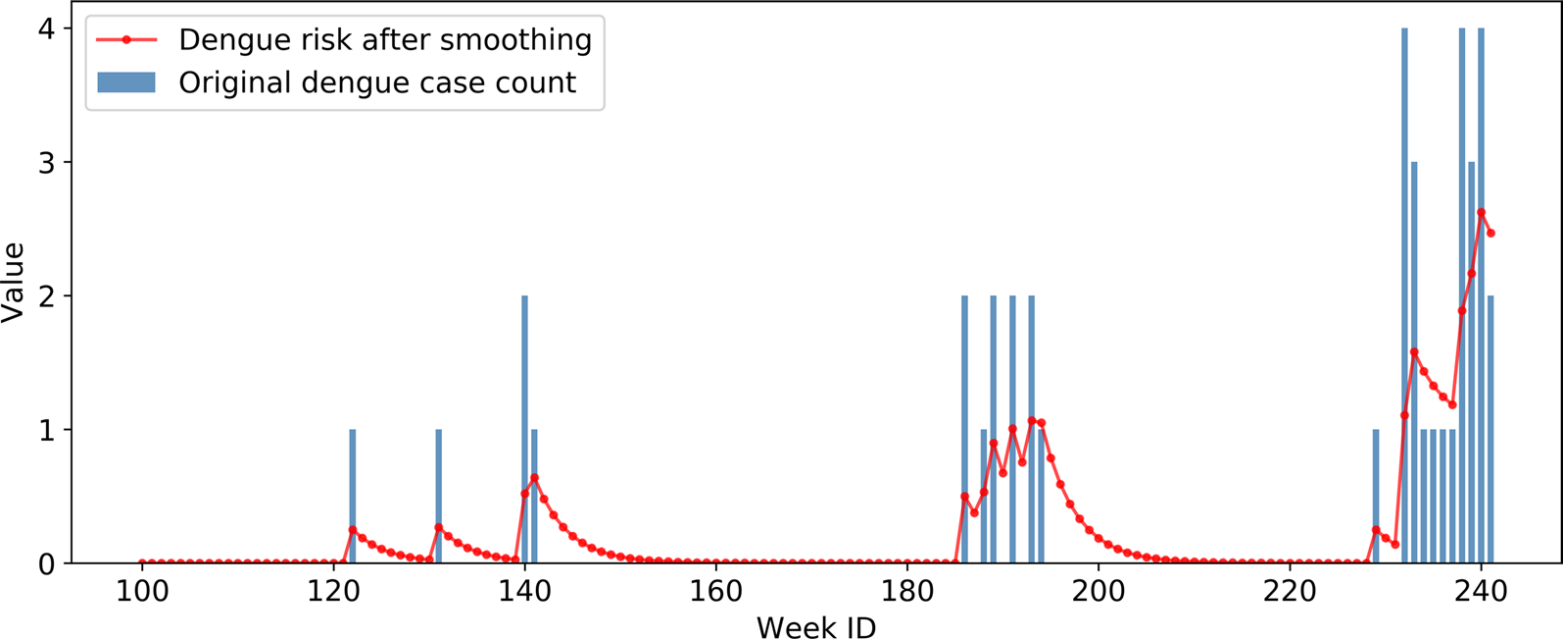
Time series of weekly local case count before and after data-smoothing

The SVM/MLP models were trained separately for 1–8 weeks ahead of dengue forecasting. The input of each model consisted of a 64-dimensional environmental feature vector and an 11-dimensional common feature vector. Each feature vector dimension was normalized to a range between zero and one using the min–max feature scaling method. As for the output of the model, because strict intervention measures were taken during the study period, the number of dengue cases in Guangzhou was very small (Fig. 3), especially at the township level, making it difficult to directly predict the local case count of each township in following weeks. To alleviate the data sparsity problem, we applied an exponential smoothing technique to the time series of weekly local case counts in each township and used smoothed value as a proxy for the real case count and the output of the model. Denoting the raw time series as {${x}_{t}$}, the smoothed time series {${s}_{t}$} is obtained using the following formulas:

1 $$s_{0} = x_{0} ,$$

2 $$s_{t} = \alpha_{s} x_{t} + \left( {1 - \alpha_{s} } \right)s_{t - 1} , t > 0,$$

where ${\alpha }_{s}$ is the smoothing factor in the range [0, 1]. Setting ${\alpha }_{s}$ as 0.25, we derived the smoothed dengue case count for each township in each week. Taking a randomly selected township as an example, Fig. 10 displays the original time series of the weekly local case count and smoothed result. The data-smoothing scheme can help retain the latent temporal patterns of dengue epidemics, and mitigate the influence of data sparseness and uncertainty caused by human intervention.

### Performance evaluation

Dengue forecasting conducted at a large spatial scale (e.g., country, state/province, and city) usually aims to provide an early warning. In contrast, a fine-grained intra-urban forecast focuses more on identifying regions with relatively higher risk in the near future, facilitating precise prevention and control despite limited resources. Therefore, in addition to measuring the Pearson correlation coefficient of the predicted and observed case counts, we also defined a “hit rate” metric to assess the forecasting performance from a spatial perspective by evaluating the ability of the model to identify high-risk urban units across the city.

Specifically, the hit rate metric calculates the proportion of dengue cases captured by the top $m$% of the predicted high-risk units during week $t$:

3 $$Hit \, rate_{t} = \frac{{N_{m, t} }}{{N_{t} }},$$

where ${N}_{m, t}$ represents the number of observed cases within the top $m$% predicted high-risk units (i.e., ranking all units from high to low according to their predicted case counts), and ${N}_{t}$ denotes the total number of observed cases within the city. A high hit rate indicates that units with higher risk during the week have been well identified by the forecasting model.

## Results

Using Guangzhou as a case study, data collected from January 26, 2015 to December 31, 2018 (a total of 167 townships × 205 weeks of samples) were used for training, and data collected from January 1 to September 22, 2019 (a total of 167 townships × 38 weeks of samples) were used for evaluation.

In this section, we first demonstrate the forecasting results from temporal and spatial perspectives, and then compare and evaluate the performance of the proposed approach quantitatively using the Pearson correlation coefficient and hit rate metric.

### Demonstrations of forecasting results from temporal and spatial perspectives

**Fig. 11:**
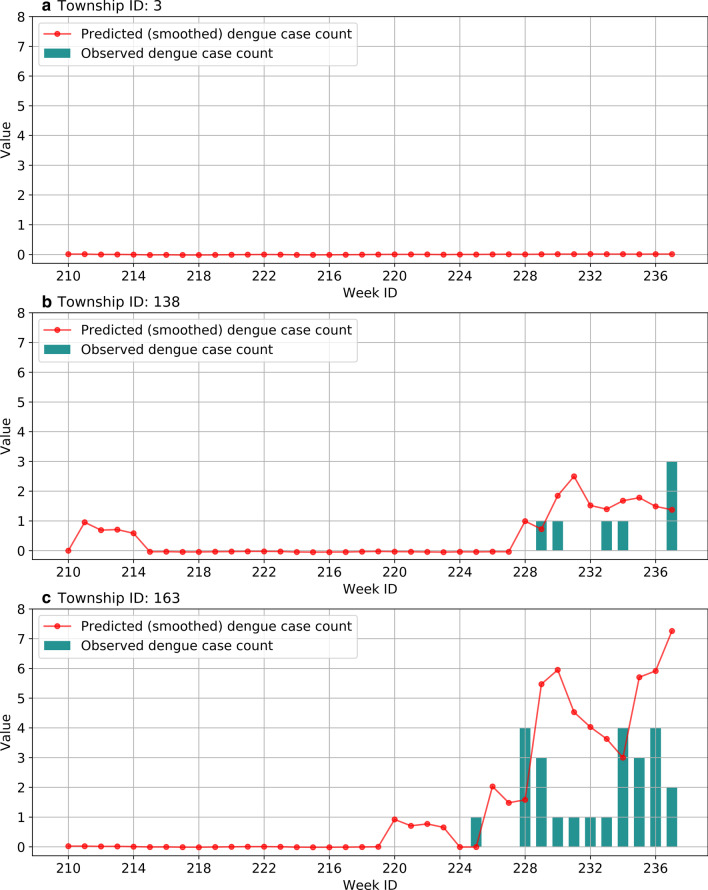
Temporal distribution of predicted case counts of three townships

From a temporal perspective, Fig. 11 presents the (smoothed) dengue case count of three townships in Guangzhou during the validation period predicted by the 1-week ahead MLP model using both common and environmental features. We can see that the predicted case count at the township level was generally parallel to the temporal trend of the actual dengue epidemic, which can serve as an early warning for preparing prevention and control measures.

**Fig. 12:**
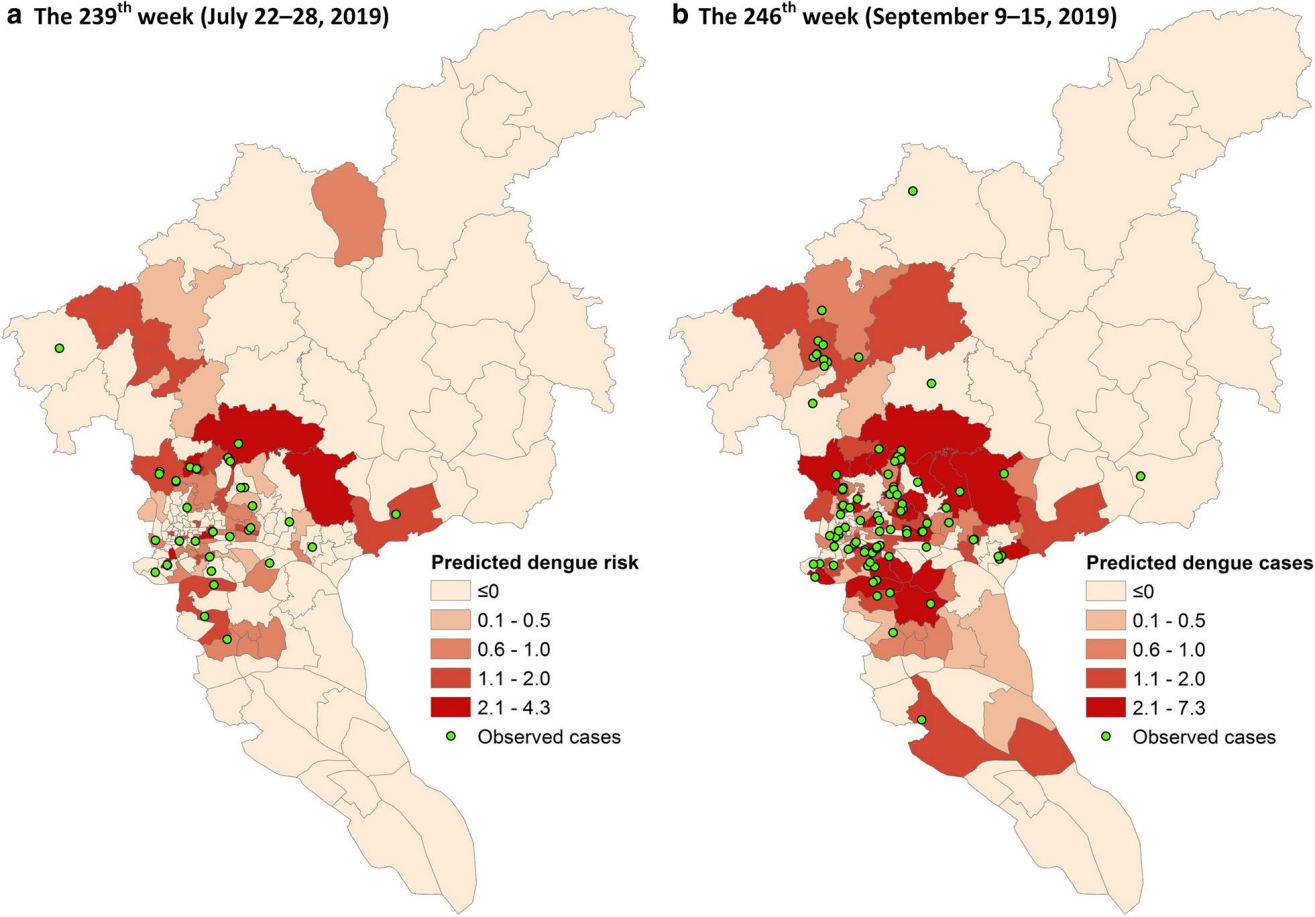
Predicted (smoothed) case counts of all townships during two different weeks. The smoothed case counts were predicted by the 1-week ahead MLP-based model using both common and environmental features

From a spatial perspective, Fig. 12 demonstrates the (smoothed) case counts of all townships across the city during two different weeks (i.e., the 239th week and the 246th week) predicted by the 1-week ahead MLP model using both common and environmental features. In the 239th week, the top 30% and 50% high-risk townships (i.e., the top 50 and 84 high-risk townships) captured 75.0% and 82.1% of the actual dengue cases, respectively. In the 246th week, the top 30% and 50% high-risk townships captured 62.5% and 78.4% of the actual dengue cases, respectively. High-risk townships can be generally identified by the proposed approach, which is potentially useful in guiding dengue prevention and control in practice.

### Performance comparison and evaluation

**Fig. 13:**
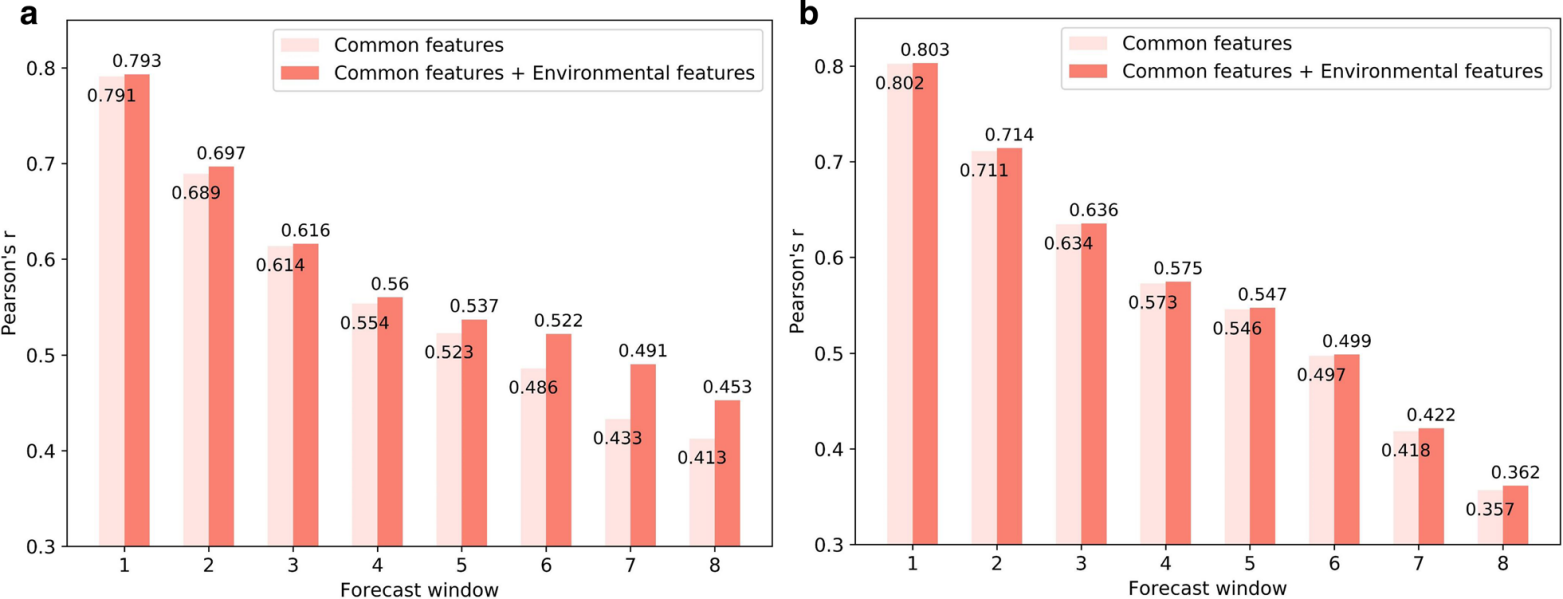
Forecasting performance evaluated by Pearson correlation coefficient. **a** MLP-based forecasting. **b** SVM-based forecasting

First, we calculated the Pearson correlation coefficients (Pearson’s r) of the predicted and observed dengue case counts. Figure 13 shows that the Pearson’s r gradually decreased with the increase in the forecast window, and the MLP models with combined environmental features outperformed those with only common features, thereby indicating the usefulness of the environmental features in dengue forecasting.

**Fig. 14:**
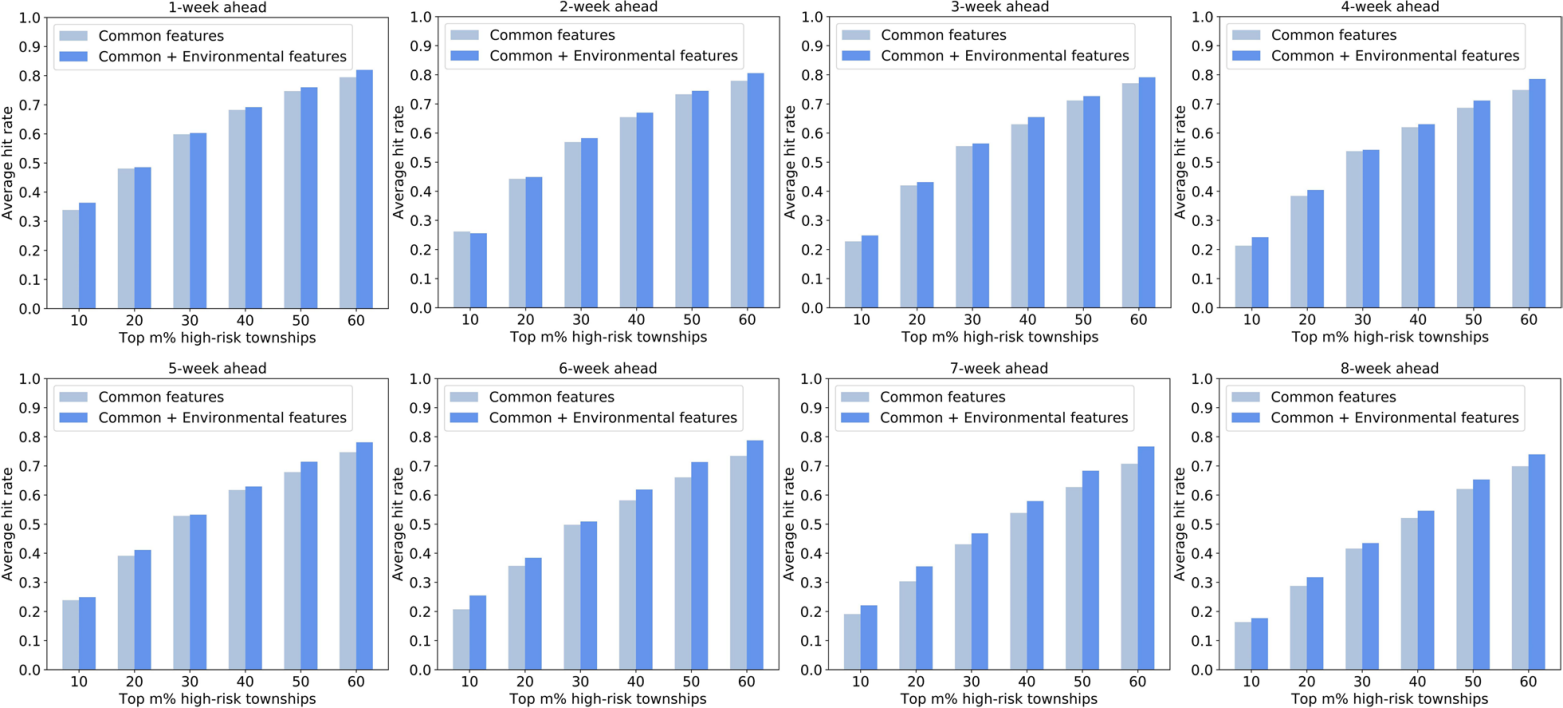
Forecasting performance of MLP-based models evaluated by the hit rate metric

**Fig. 15:**
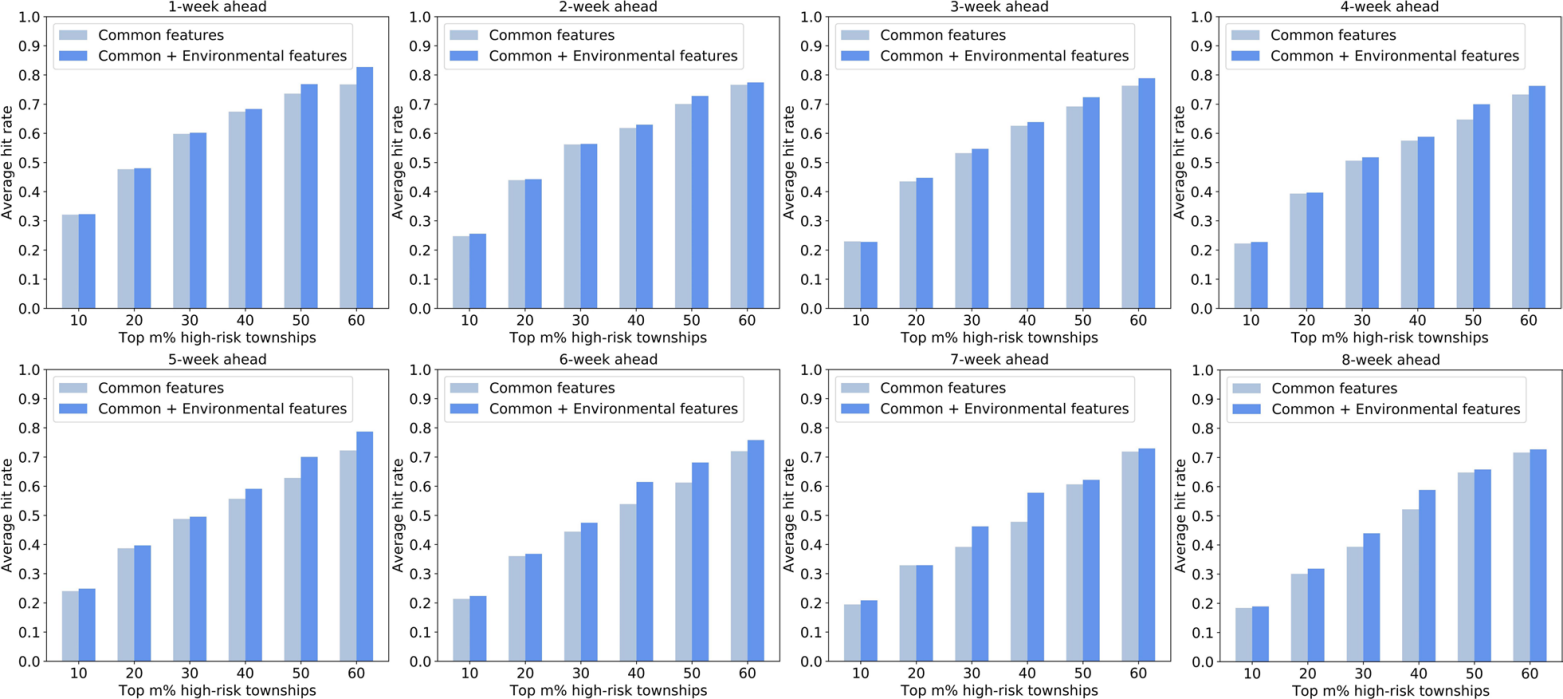
Forecasting performance of SVM-based models evaluated by the hit rate metric

Second, we used the hit rate metric to measure the ability of the models to identify high-risk townships across the city. Figures 14, 15 show the average hit rates during the defined outbreak period. We can see that the top 30% of the predicted high-risk townships captured approximately 50–60% of the dengue cases across the city. Such risk maps can help guide precise dengue prevention and control in urban spaces. In addition, the results indicated that models using both common and environmental features as inputs behaved better than those using only common features, thereby further proving that incorporating urban environments measured from street-view images effectively facilitates dengue forecasting.

## Discussion

As a mosquito-borne infectious disease, the incidence rate of DF is highly related to local meteorological and environmental conditions. As current studies have mainly been conducted at large spatial scales, only meteorological variables have been extensively used for dengue forecasting. However, environmental suitability is also a vital factor in the dengue incidence, especially at a fine-grained intra-urban scale. Some local areas (e.g., dustbins, flowerpots, building sites, etc.) typically provide more ideal conditions for mosquito breeding and survival than others, even under the same suitable weather conditions. Therefore, combining local environments and meteorological factors can theoretically help enhance fine-grained intra-urban dengue forecasting.

Historically, measuring urban physical environments has been laborious work, while the emergence of street-view images and the development of deep learning techniques enable automatic feature extraction of local environments across a city. Taking advantage of the promising data source and advanced techniques, this study proposes a framework for facilitating fine-grained intra-urban dengue forecasting by incorporating local environments measured from street-view images. First, a pre-trained PSPNet model was applied to extract the outdoor scene features of the street-view images, and the feature vectors of images collected from the same unit were averaged as unit environmental feature vectors. The environmental feature vectors were then combined with the commonly used features (e.g., temperature, rainfall, past cases) to enhance the supervised learning-based dengue forecasting models. The case study conducted at the township level for dengue forecasting in Guangzhou City indicated that models using both common and environmental features behaved better than those using common features alone, proving that incorporating urban environments measured from street-view images can help facilitate dengue forecasting in small-scale urban areas.

We summarized the highlights of this study from the following two perspectives.

1. Most existing dengue forecasting studies have been conducted at large spatial scales such as the national, sub-national, and city levels, whereas this study focused on fine-grained intra-urban areas, which can help identify high-risk regions for precise dengue prevention and control.
2. Considering that local environments significantly influence vector suitability, we introduced emerging street-view images and advanced deep learning techniques to extract environmental features, which can help enhance dengue forecasting at small spatial scales. To the best of our knowledge, this is the first attempt to apply this promising data source to dengue forecasting.

Our research has some limitations that can be addressed in future work. First, the performance improvements made by environmental features were not significant in our study. One important reason is that the dengue case data during the study period are too sparse (especially at the township level), rendering forecasting very difficult. However, considering that both the hit rate metric and Pearson’s correlation coefficient are improved for all forecasting windows, we still conclude that the environmental features are effective in enhancing fine-grained dengue forecasting. Second, it is difficult to obtain dynamic population and street-view images at one-week intervals. Considering that Guangzhou is a well-developed city, we chose to use the population data of 2017, representing the population of 2015–2019, and the most recent street-view image data provided by Baidu API. Third, the dengue case data used in this study are dependent on notifiable data, while asymptomatic cases and immunity levels were not considered. Finally, because street-view vehicles can only scan horizontal views along linear public roads, street-view image data are still inadequate for depicting the overall conditions of a region. Therefore, we suggest that the bird-eye view images collected by drones (if available) can be used together with street-view images to better depict the environments of the regions.

## Conclusions

Considering that emerging street-view images are promising data for depicting physical environments, this study proposed a fine-grained intra-urban dengue forecasting approach by integrating physical environments measured from street-view images. The results show that models integrated with environmental features behaved better than those using traditional features alone, proving that our proposed framework effectively incorporates environmental factors and facilitates intra-urban dengue forecasting at small spatial scales.

## Data Availability

Data except the dengue cases are available from the corresponding author upon reasonable request. The georeferenced dengue case data described in the manuscript cannot be shared directly because the data reveal patient’s locality and hence compromise patient privacy. However, data are available from the Guangdong Provincial Center for Disease Control and Prevention (contact via gdcdcp@cdcp.org.cn) for researchers who meet the criteria for access to confidential data.

## Abbreviations

DF: Dengue fever
MLP: Multilayer perceptron
SVM: Support vector machine
NDVI: Normalized difference vegetation index
APIs: Application programming interfaces
CDC: Center for Disease Control and Prevention
OSM: OpenStreetMap
